# The Association Between Grandchild Care and Biological Aging Among Middle-Aged and Older Adults in China

**DOI:** 10.1093/geroni/igae059

**Published:** 2024-06-17

**Authors:** Donghong Xie, Jiwen Wang

**Affiliations:** School of Political Science and Public Administration, Shandong University, Qingdao, China; Centre for Quality of Life and Public Policy Research, Shandong University, Qingdao, China; Institute of Population and Social Development, Shandong Academy of Social Sciences, Jinan, China

**Keywords:** Biomarkers, Gender difference, Grandchild care, Role strain, Shared caring

## Abstract

**Background and Objectives:**

Substantial evidence documents grandchild care is associated with self-reported health, life satisfaction, and depressive symptoms among middle-aged and older adults. However, little is known about the relationship between grandchild care and biological aging, especially in China, which emphasizes the unique cultural value of family. The current study sheds light on the biological consequence of grandchild care by examining the link between grandchild care and biological aging among middle-aged and older adults in China, and how gender and spousal involvement in caregiving affect this link.

**Research Design and Methods:**

In a representative sample of Chinese adults aged 45–80 from the third wave of China Health and Retirement Longitudinal Study in 2015 (*n* = 3,384), we calculate biological age using Klemera–Doubal Method, and Ordinary Least Square models are used to examine the correlation between grandchild care and biological aging.

**Results:**

High intensity of involvement in grandchild care is related to biological aging, and caring for grandchildren alone predicts greater biological aging. Compared with grandfathers, grandmothers lose more from grandchild care regardless of whether their husbands are involved in the care.

**Discussion and Implications:**

Providing grandchild care should be a way to cope with age-related role discontinuity or loss, rather result in extra stress or burden for grandparents. Reducing the intensity of caregiving or increasing family support may attenuate the extent of biological aging.

**Translational Significance:** We extend the literature on grandchild care to field of biology. High intensity of grandchild care is associated with greater biological aging among middle-aged and older adults in China. Shared care alters the association between grandchild care and biological aging for grandfathers, however, grandmothers lose more from grandchild care regardless of whether their husbands are involved in the care. This suggests an urgent need to increase family support or extend policies to alleviate the caregiving responsibility for a large proportion of Chinese grandparents especially grandmothers who, in addition to facing their own aging challenges, have to care for grandchildren.

There are two objective types of age in one’s lifetime: chronological age and biological age. The former is absolute and moves in lockstep with the calendar time, although the latter is relative and increases at a stochastic nonlinear rate in time that is a clock that might occasionally move backwards (Huang et al., 2017). Individuals of the same chronological age may thus differ in biological age. As suggested by Levine and Crimmins (Levine & Crimmins, 2018), if an individual is 40 years old in calendar and 50 years old in biology, it may suggest that he/she is aging at an accelerated rate. Previous studies have shown that biological age, as a reliable predictor of both morbidity and mortality, reflects more accurately the aging rate of human in relation to functional declines and deteriorating health than chronological age (Levine, 2013; Schaefer et al., 2016).

Several studies conducted in Western populations have shown that biological age is malleable, subject to environmental factors, genetic differences, and some level of stochasticity (Kirkwood, 2002; Levine & Crimmins, 2018). Recent research shows that social stress, in the form of traumatic events, job strain, everyday stressors, and discrimination, accelerates aging of the immune system, potentially increasing one’s risk of cancer, cardiovascular disease, and reducing efficacy of vaccines (Klopack et al., 2022). Among the array of stressors that middle-aged and older adults may confront in their daily lives, grandchild care represents a common but subtle source of stress, especially in Chinese context due to the unique cultural values and societal norms of family, which place a high value on the collective wellbeing of their extended families and value family solidarity, harmony, and continuity (Xu, 2019). Grandparents involved in grandchild care on a regular basis is often considered as an obligation and a family adaptive strategy that eases the burden on adult children and obtains emotional or material support from their adult children. In Western countries, however, societal norms tend to favor a downward flow of intergenerational support from parents to children rather than the reverse. Grandparents are thus used to providing only supplementary help in grandparenting (Jendrek, 1993; Pruchno, 1999), unless their adult children are divorced, substance abusers, or incarcerated (Burnette et al., 2012; Goodman & Silverstein, 2002). The health and aging consequences of grandchild care may deserve further study in the Chinese context due to the differences across cultural norms.

Although a growing literature demonstrates the links between grandchild care and middle-aged and older adult’s self-reported health (Di Gessa et al., 2016; Ku et al., 2013), life satisfaction (Ku et al., 2013; Silverstein et al., 2006; Xu, 2019) and depressive symptoms (Bordone & Arpino, 2019; Cong & Silverstein, 2008), these measures may not adequately capture differences of health and aging between groups. Previous research has shown that health differences between higher- and lower-educated people, as measured by biomarkers, are underestimated by self-assessed health measures (Dowd & Zajacova, 2010), and the predictive power of poor self-assessed health for mortality decreases with age (Zajacova & Woo, 2016). In fact, biological changes caused by grandchild care may be weak and gradual, and therefore difficult to be detected. Instead, biological age constructed from biomarker set could capture more accurately the subtle and progressive changes in one’s functioning and health caused by caring for grandchildren.

## Grandchild Care and Biological Age

According to role enhancement and role strain theories, caring for grandchildren may be either rewarding or stressful for individuals. Role enhancement refers to the psychological and physiological benefits of occupying multiple roles (Sieber, 1974). Social roles help individuals gain opportunities for social support and satisfaction, and caring for grandchildren is often considered as a “productive role.” Based on this theory, empirical literature has shown that caregiving to grandchildren benefits grandparents psychologically as it strengthens ties between grandparents, parents, and grandchildren, provides them with a sense of purpose and meaning in life, and enhances feelings of self-efficacy and self-esteem (Hughes et al., 2007). The boost in psychological wellbeing may translate into better physiological health (Di Gessa et al., 2016; Ku et al., 2013), less hypertension (Xu, 2019), and increased longevity (Hilbrand et al., 2017) for those who care for grandchildren, compared with noncaregivers. Therefore, from the perspective of role enhancement, grandchild care involves honor and fulfillment, although the other theory argues oppositely. Role strain theory suggests that social roles involve responsibilities that individuals generally want to fulfill if the role is meaningful, although some individuals may perceive it hard to do so when role obligations exceed one’s physical and mental resources or capacities, which is called role strain (Pearlin, 1980). Although caring for grandchildren on a regular basis could be a labor of love, it could also be a chronic stressor, especially caring for left-behind children alone in rural areas, reflecting more role tension than role enhancement related to fulfillment. Since caring for grandchildren requires more routine parenting tasks, it limits grandparents’ time and opportunities to participate in leisure and recreational activities (Jendrek, 1993), social participation (Pruchno, 1999), self-care and medical care (Baker & Silverstein, 2008). As a result, it may incur high levels of burden and stress to grandparents, and consequently lead to wear and tear on the body and premature aging (Forrester et al., 2019, 2022a, 2022b), earlier onset of chronic disease (Lee et al., 2003), lower functional status (Fuller-Thomson, 1999), lower active life expectancy, and earlier mortality (Geronimus et al, 2001).

In fact, the biological outcome associated with grandchild care may vary by the level of caregiving intensity and context. According to the stress process model (Pearlin, 1989), role strain or role overload caused by intensive caregiving may be harmful to biological health. Long-term higher caregiving intensity compounded by other stressors, such as role expectation and family contradiction, may lead to body wear down and rapid aging (Keller et al, 2019), which has been demonstrated by using multiple measures including allostatic load (Geronimus et al, 2006), epigenetic age (Liu et al., 2019), and biological age (Forrester et al., 2019, 2022a, 2022b). That is, when caregiving task constitutes a chronic, unrelenting and uncontrollable stressor, multiple biological systems have suffered from cumulative damage in adapting to the stressors, increasing disease risk and vulnerabilities of aging among custodial grandparents (Charles, 2010; Roepke et al., 2011).

## Contexts of Caregiving: Gender Norm and Spousal Involvement

As suggested by ecological theory of human development (Bronfenbrenner, 1986), both the micro environment (eg, family and friends) and the macro environment (eg, broader social and cultural context) have an impact on individual development. It is therefore reasonable to speculate that the contexts of grandparenting may shape or influence caregiving experience and health outcomes. Gender context is an important aspect in considering the role strain or role enhancement when we examine the health and aging outcomes in grandparenting in China (Xu, 2019). On the one hand, caregiving and family responsibilities are often assigned to women, although financial breadwinner responsibilities are assigned to men. Grandmothers thus take on more intensive caregiving responsibilities, such as feeding, bathing, and dressing, although grandfathers tend to play the role of fun-seekers and playmates (Lo & Liu, 2009; Xie & Xia, 2011). Meanwhile, there are also indications that grandparental care differs by gender: partnered grandfathers are more likely to care for grandchildren, although partnership status makes no difference to grandmothers’ care propensity (Horsfall & Dempsey, 2013). This suggests grandfathers commonly rely on their wives to facilitate their time with grandchildren, and are less involved with extended family if they are divorced or widowed. In this case, grandmothers might receive less help from their husbands than grandfathers do from their wives, which may cause females to experience more role strain and role stress. However, on the other hand, grandmothers may have acquired more skills, resources, and coping strategies throughout their lives to properly care for their grandchildren than grandfathers, and grandmothers’ role are further reinforced by a greater sense of satisfaction or fulfillment in caring for grandchildren, which may alleviate role burden or role stress. In contrast, grandfathers may experience additional role strain or even social stigma because of the departure from traditional gender norm if they are heavily involved in caregiving. Chinese grandfathers experience a faster decline in self-rated health than grandmothers when they involve in high-intensity grandchild care. (Chen & Liu, 2012).

In fact, the discussion above has already alluded to the significance of spousal involvement in understanding the health outcomes of grandchild care. Spousal support, as a kind of family support, may serve as a protective factor and mitigate the negative health effects of caregiving, as postulated according to the stress-buffering mechanism. Positive spousal support plays an important role in addressing caregiving distress, whereas the strain from families may increase depressive symptoms and loneliness (Tang et al., 2016). Grandparents who are raising grandchildren may be able to prevent or manage stress with the support of family network, especially with the involvement of spouses. When a family is cohesive and both husband and wife participate in caregiving, the family not only provides emotional help, such as advice, encouragement and emotional substance, but also reduces individual time spent on caregiving and relieves the fatigue of caregiving. Therefore, grandparents involved in shared care may be more accustomed to fulfilling intensive caregiving obligations and coping with associated physical and mental stress than those who care for their grandchildren alone.

## The Present Study

Following from key propositions of role enhancement, role strain, and stress process model, this study goes beyond self-reported health outcomes by using accurate biomarker information to examine whether grandchild care is related to biological aging and how gender and spousal involvement in caregiving shape this relationship among middle-aged and older adults in China. We posit three hypotheses:

(1) Greater involvement in the caregiving role, indicated by spending more time caring for grandchildren or looking after more grandchildren, may accelerate biological aging of grandparents (Hypothesis 1).
(2) There is a gender difference between the association of grandchild care with biological aging, and grandmothers experience greater biological aging than grandfathers (Hypothesis 2).
(3) The association between grandchild care and biological aging varies by whether spouse involves in caregiving or not. Specifically, compared with caring for grandchildren alone, shared caregiving with couple is expected to produce less strain and have lower risk of biological aging (Hypothesis 3).

## Method

### Study Participants

Data are from the China Health and Retirement Longitudinal Study (CHARLS), an ongoing nationally representative, longitudinal survey of people aged 45 years or older and their spouses of any age collecting respondents’ demographic information, family transfer, health status, and functioning. Using a multistage probability sampling design, CHARLS covers 14,000 households in 150 counties and 28 provinces. The first, second, third, and fourth national waves were completed in 2011, 2013, 2015, and 2018, respectively, with the blood biomarkers only available for 2011 and 2015. The first wave of blood collection lagged the survey by half a year due to the difficulty of working in the field, and some batches of blood in first wave underwent temperatures above freezing during transportation, which might affect the accuracy and validity of biomarkers. In this study, we thus mainly use data from the third wave (*n* = 21,097), with 13,419 respondents providing venous blood, accounting for 63.6% of the sample. A more detailed description of the venous blood-based biomarkers design, sampling procedure, and rationale in the third wave appears elsewhere (Chen et al., 2019).

Considering the sample representativeness and the survival selection effect of the older adults, we exclude those below aged 45 years (*n* = 669), over aged 80 years (*n* = 388), and nonfasting blood samples (*n* = 1,858). We then exclude those with missing biomarker (n = 341), and biomarker outliers by computing gender-specific mean and standard deviation, with more than 5 standard deviations excluded (Kwon & Belsky, 2021) (male = 213, female = 249). In total, there are 9,701 adults with valid biomarker measures. We match the valid blood data based on ID to obtain their grandparenting status, sociodemographic data, and information on health status and cognitive function. Notably, when respondents have any grandchildren under the age of 16, CHARLS would ask them if they spend time caring for grandchildren, otherwise skipping the question. Therefore, the sample size is reduced after matching, with 3,384 respondents providing valid biomarkers, grandparenting status responses and with no missing values on covariates in 2015.

### Measures

#### Biological aging

Biological age is calculated using the Klemera and Doubal Method, which is through a mathematical model based on minimizing the distance between *n* regression lines and *n* biomarker points, within an *n* dimensional space of all biomarkers. The equation used to calculate biological age combines information on the respondents’ measured biomarker values (*x*_*i*_), as well as the slope (*k*_*i*_), intercept (*q*_*i*_), and root mean squared error (*s*_*i*_) from the equation of each biomarker regressed on chronological age. The *s*_*BA*_ is a scaling factor equal to the square root of the variance in chronological age explained by the biomarker set. CA is chronological age. More details could be found in the article by Klemera and Doubal (Klemera & Doubal, 2006).


$$\begin{array}{l} BA=\frac{\overset{n}{\underset{i=1}{\sum }}\left(x_{i}-q_{i}\right)\left(\frac{k_{i}}{s_{i}^{2}}\right)+\frac{CA}{s_{BA}^{2}}}{\overset{n}{\underset{i=1}{\sum }}{\left(\frac{k_{i}}{s_{i}}\right)}^{2}+\frac{1}{s_{BA}^{2}}} \end{array}$$

As there is gender difference in the association of different biomarkers with chronological age (Kwon & Belsky, 2021; Zhong et al., 2020), we construct gender-specific biological age using different biomarkers. There are 18 available biomarkers measures of aging in our sample, and the selected biomarkers and descriptions are listed in Supplementary Table S1. The selection procedure is to removing biomarkers with relatively smaller contribution to age estimation and strong intercorrelations. For males, eight biomarkers out of the 18 are nonsignificant or the correlation coefficient is less than 0.1 with chronological age, hence are removed from the final list. Variance inflation factors (VIFs) of the selected 10 biomarkers are 1.6, and the correlation coefficients between them are mostly less than0.3. The 10 selected biomarkers for males cover a gamut of biological functioning in three domains: (a) Vascular function (Systolic Blood Pressure and Diastolic Blood Pressure); (b) Immune function (Hematocrit and Platelets); (c) Metabolic function (Hemoglobin, Glycated Hemoglobin, Creatinine, Blood Urea Nitrogen, Cystatin C, and Triglyceride). For females, seven biomarkers are removed from the 18 biomarkers, and VIFs of the selected 11 biomarkers are 1.0, having correlation coefficients between them mostly less than0.4. The 11 selected biomarkers cover biological functioning in three domains: (a) Vascular function (Systolic Blood Pressure); (b) Immune function (C-Reactive Protein and Mean Corpuscular Volume); (c) Metabolic function (Creatinine, Blood Urea Nitrogen, LDL Cholesterol, Total Cholesterol, Uric Acid, Cystatin C, Glycated Hemoglobin, and Glucose).

Biological aging is used to define the difference between biological age and chronological age, which captures the relative difference between the two objective ages and reflects whether the respondent is biologically older or not. A positive value indicates that a person is biologically older than one’s chronological age, whereas a negative value indicates that a person is biologically younger.

#### Grandchild care

CHARLS asks respondents whether they or their spouses had taken care of their grandchildren last year. If a respondent answers “yes,” he or she would be asked about the weeks and hours per week of each child’s children provided by the respondent and his/her spouse, respectively. It is worth noting that even if the respondent answers that he/she or spouse has cared for grandchildren in the past year, the amount of time the respondent provided for grandchildren in the second question could be zero, as the grandchildren may be cared for entirely by his/her spouse.

We are interested in the association between grandchild care and biological aging. Therefore, we use the two questions above to construct another two categorical variables reflecting one’s caregiving intensity: (a) the average hours per week on grandchild care (ie, the first answer is “yes” and the total amount of time he/she provided is positive in the second question). We further divide the time on grandchild care into zero hours (noncaregivers), low-intensity care (<10 hr per week last year), medium-intensity care (between 10 hr and 40 hr per week last year), and high-intensity care (40 hr and above per week last year). The category is based on the following reasons. First, the legal working hour in China is 8 hr every working day and 40 hr every week, so caring for grandchildren for 40 hr or more per week is thus equivalent to a high-intensity full-time job. Second, the “threshold” for caregiving intensity in the international literature is usually 10 hr per week (Carmichael & Charles, 2003a, 2003b; Ettner, 1995). Caring for less than 10 hr a week involves a less degree of responsibility, and requires regular but relatively undemanding “a bit help,” and grandparents have enough time to enjoy personal leisure activities and self-care. (b) the number of grandchildren caring for last year (none = 0, one grandchild = 1, two grandchildren and above = 2). Although the total amount of time that grandparents spent has taken into account the number of grandchildren being cared for, we recognize that the number of grandchildren being cared for is still another important indicator affecting the role of grandparents, especially when there is usually age difference among grandchildren. Due to the different needs of age-various grandchildren, grandparents may spend more nonsharable energy caring for two or more children.

In order to estimate the impact of spousal participation in caregiving, we also distinguish the four different types of grandchild care: no grandchild care (the first answer is “no”); cared by oneself alone (positive hours provided by oneself and zero hours provided by his/her spouse); cared by spouse alone (positive hours provided by his/her spouse and zero hours provided by oneself); and cared by both (both positive hours by oneself and his/her spouse).

#### Covariates

In regression models, we adjust for sociodemographic information, family context, and health-related characteristics correlated with both grandparenting and biological aging. Measures include chronological age, age-squared, gender (male = 1; female = 0), *hukou* (rural = 1; urban = 0) and education level (illiterate = 1; completed primary school = 2; completed middle school or above = 3). We control for marital status (married = 1; divorced or separated = 2; widowed = 3), because according to social obligation perspective, individuals might eat more regularly, choose healthier meals, and follow the health-promoting norms within a relationship, which could influence biological aging. Models also adjust for parental mortality (whether each parent is alive), as family members may share genetics that influence aging (Donnelly et al, 2020). Measures of health characteristics include smoking status (yes = 1; no = 0), whether the respondent drinks alcohol (yes = 1; no = 0), body mass index (BMI: underweight = 1; normal = 2; overweight = 3; obesity = 4), self-reported health (from 1 to 5: poor = 1, excellent = 5) and number of functional limitations (number of activities of daily living limitations: from 0 to 6; number of instrumental activities of daily living limitations: from 0 to 7). We also adjust for diagnosed conditions that are directly related to biological aging, including hypertension, diabetes, hyperlipemia, heart problems, stroke, and cancer. Finally, all models control for the province variable due to the substantial geographic variations in social development, healthcare, and environmental conditions between regions, which might influence individuals’ health and aging process.

### Analytic Strategy

We first calculate descriptive statistics, including the proportion of grandchild care, grandchild care intensity, and biological age among middle-aged and older adults in China, overall and by gender. To assess whether the intensity and number of grandchild care are associated with biological aging, we then estimate two linear regression models where they are included one at a time, controlling for sociodemographic information, family context, and health-related characteristics.

Next, to evaluate whether the relationship between grandchild care and biological aging vary by gender, we create interaction terms of caregiving intensity or the number of grandchild care with gender, controlling for all covariates. Furthermore, to test the hypothesis regarding the effect of spousal involvement, we reestimate models by adding grandchild care types and evaluate whether different types of grandchild care on biological aging are consistent across genders.

Finally, four sensitivity analyses are conducted. (a) We use biological aging proportional difference score instead of biological aging to reestimate the association. (b) To mitigate the possible bias due to dual burden of caregiving (caring for grandchildren and their own spouses, simultaneously), we repeat analyses excluding cases whose spouse loses self-care ability. (c) As living arrangements may not happen randomly and could be affected by work and childcare arrangements (Chen et al., 2011), we distinguish the effects of residential and nonresidential grandchild care on biological aging in grandparents. (d) Although we control for many sociodemographic and health characteristics, it is possible that unobserved factors correlated with grandparenting and biological aging bias our estimates. Because 265 respondents among 738 respondents changed grandparenting status in our sample between 2011 and 2015, we are able to explore this possibility. Analyses were completed using Stata15.0 and R Studio 4.2.

## Results

### Sample Description

General characteristics of demographic factors, caregiving status, and biological aging are presented in Table 1. Overall, both chronological age and biological age have means of 62.1 years as expected, although the standard deviation is larger for biological age (9.33) than for chronological age (8.19). Nearly half (46.78%) of respondents took care of their grandchildren last year, with 10.85% providing low-intensity caregiving, 12.65% providing medium-intensity caregiving, and 23.29% providing high-intensity caregiving. In terms of the number of caregiving, nearly 40% of respondents care for one grandchild, and only 7% care for two or more grandchildren at the same time. In addition, 13.15% of respondents care for grandchildren alone, 33.63% care for grandchildren with their spouse, and 2.81% of respondents leave it for their spouse alone. However, when caregiving types are examined by gender, significant variations emerge as shown in Table 1. Consistent with the traditional gender norm, 21.81% of grandmothers report caring for grandchildren alone, although only 4.59% of grandfathers report caring for grandchildren alone.

**Table 1.** Summary Statistics

Variable	All (*N *= 3,384)		Male (*n* = 1,701)		Female (*n* = 1,683)	
	Mean (*SD*)	%	Mean (*SD*)	%	Mean (*SD*)	%
Biological age	62.1 (9.33)		63.3 (8.51)		60.8 (9.94)	
Age	62.1 (8.19)		63.3 (7.69)		60.8 (8.49)	
Caregiving intensity						
No care		53.22		56.38		50.03
Low		10.85		10.70		10.99
Medium		12.65		12.05		13.25
High		23.29		20.87		25.73
The number of caregiving						
None		53.22		56.38		50.03
One grandchild		39.75		36.21		43.32
Two grandchildren and above		7.03		7.41		6.65
Caregiving types						
No care		50.41		50.97		49.85
Caregiving alone		13.15		4.59		21.81
Caregiving by spouse alone		2.81		5.41		0.18
Caregiving by couples		33.63		39.04		28.16
Male		50.27				
Education level						
Illiterate		23.70		10.11		37.43
Primary school		44.24		48.32		40.11
Middle school and above		32.06		41.56		22.46
Rural *hukou*		82.71		81.01		84.43
Marital status						
Married		80.20		88.12		72.19
Divorced or separated		1.00		1.29		0.71
Widowed		18.79		10.58		27.09
Mother alive		18.11		15.23		21.03
Father alive		9.54		7.64		11.47
Smoking status		29.79		53.56		5.76
Drinks alcohol		35.43		54.85		15.81
Body mass index						
Underweight		5.26		5.82		4.69
Normal		37.77		44.21		31.25
Overweight		21.25		19.69		22.82
Obesity		35.73		30.28		41.24
Number of ADL limitations	0.39 (0.93)		0.34 (0.89)		0.45 (0.96)	
Number of IADL limitations	1.49 (1.61)		1.19 (1.52)		1.79 (1.64)	
Self-reported health						
Poor		5.08		4.53		5.64
Fair		21.10		20.34		21.87
Good		52.28		51.73		52.82
Very good		10.99		12.35		9.63
Excellent		10.55		11.05		10.04
Diagnosed diseases	0.88 (1.04)		0.82 (1.03)		0.94 (1.06)	

*Notes*: ADL = activities of daily living; IADL = instrumental activity of daily living; *SD* = standard deviation.

### Associations of Grandchild Care With Biological Aging

Table 2 presents results from ordinary least square regressing biological aging on grandchild care, adjusting for additional sociodemographic information, family context, and health-related characteristics. Model 1 estimates whether caregiving intensity influences biological aging, and Model 2 distinguishes the effect of the number of grandchild care.

**Table 2.** Associations of Grandchild Care With Biological Aging

Variable	Model 1 (*n* = 3,384)		Model 2 (*n* = 3,384)	
	*β*	*SE*	*β*	*SE*
Caregiving intensity (Ref: No care)				
Low	0.153	(0.255)		
Medium	0.216	(0.235)		
High	0.512**	(0.191)		
The number of caregiving (Ref: None)				
One grandchild			0.345*	(0.163)
Two grandchildren and above			0.345	(0.303)
Age	−0.098	(0.137)	−0.101	(0.138)
Age-squared	0.001	(0.001)	0.001	(0.001)
Male	0.349+	(0.204)	0.343+	(0.204)
Education level (Ref: Illiterate)				
Primary school	−0.071	(0.223)	−0.071	(0.223)
Middle school and above	0.044	(0.257)	0.047	(0.257)
Rural *hukou*	0.374+	(0.208)	0.370+	(0.208)
Marital status (Ref: Married)				
Divorced or separated	0.795	(0.738)	0.786	(0.738)
Widowed	−0.269	(0.234)	−0.274	(0.234)
Mother alive	0.046	(0.202)	0.050	(0.202)
Father alive	−0.173	(0.263)	−0.182	(0.264)
Smoking status	0.199	(0.176)	0.200	(0.176)
Drinks alcohol	−0.453**	(0.159)	−0.454**	(0.159)
Body mass index (Ref: Normal)				
Underweight	0.148	(0.339)	0.143	(0.340)
Overweight	0.527**	(0.202)	0.522**	(0.202)
Obesity	1.270***	(0.190)	1.268***	(0.190)
Number of ADL limitations	−0.112	(0.112)	−0.115	(0.112)
Number of IADL limitations	0.093	(0.070)	0.093	(0.070)
Self-reported health (Ref: Poor)				
Fair	−1.161*	(0.455)	−1.156*	(0.456)
Good	−1.212**	(0.453)	−1.210**	(0.454)
Very good	−1.148*	(0.495)	−1.159*	(0.495)
Excellent	−1.224*	(0.509)	−1.223*	(0.509)
Diagnosed diseases	0.783***	(0.085)	0.783***	(0.086)
Province	controlled		controlled	
_cons	3.347	(4.224)	3.469	(4.235)
*R*-squared	0.0937		0.0931	

*Notes*: ADL = activities of daily living; IADL = instrumental activity of daily living; *SE* = standard error. Standard errors, clustered at the household level, are shown in parentheses.

+*p* < .1. *p < .05. **p < .01. ***p < .001.

As shown in Table 2, high-intensity caregivers are estimated to experience greater biological aging relative to noncaregivers (*p* < .01) after equalizing control variables or risk factors for biological aging. That is, for any grandparent, providing high-intensity caregiving for grandchildren is associated with 0.512 years increase in biological aging. No significant difference is observed between noncaregivers and low-intensity, or medium-intensity care providers. Furthermore, the number of grandchildren care is also associated with biological aging, with caring for one or two and above grandchildren increasing biological aging by 0.345 years compared with noncaregivers.

Some relationships between the covariates and biological aging are worth noting. Greater biological aging is found among males and rural respondents, whereas educational and marital status are not significantly associated with biological aging. This is in contrast to health-related characteristics, where respondents having higher self-reported health are likely to have slower biological aging, and respondents with more diagnosed diseases have greater biological aging. Compared with normal BMI, overweight and obese respondents have 0.53 years and 1.27 years increase in biological aging, respectively. However, contrary to expectations, drinking alcohol is associated with slower biological aging. That may be because moderate alcohol consumption reduces the likelihood of frailty (Shah & Paulson, 2016), and lowers risks of coronary artery disease, diabetes mellitus, congestive heart failure, and stroke (Rimm et al, 1999). Besides, the extent of biological aging varies among provinces, with the lowest biological aging in Beijing and Shanghai.

### Gender Difference Between Grandchild Care and Biological Aging

Due to the different hypothesis derived from role strain and role enhancement theories, we expect grandfathers and grandmothers to react differently to the role of grandchild care. We thus evaluate how gender moderates the effects of grandchild care by performing gender interaction models, adjusting for all covariates. The results are shown in Table 3.

**Table 3.** Gender Heterogeneity Between Grandchild Care and Biological Aging

Variable	Model 3 (*n* = 3,384)		Model 4 (*n* = 3,384)	
	*β*	*SE*	*β*	*SE*
Caregiving intensity (Ref: No care)				
Low	0.385	(0.420)		
Medium	0.636+	(0.375)		
High	0.894**	(0.297)		
Male	0.673**	(0.253)	0.675**	(0.253)
Caregiving intensity (Ref: No care) # Gender				
Low # male	−0.438	(0.509)		
Medium # male	−0.833+	(0.459)		
High # male	−0.783*	(0.370)		
The number of caregiving (Ref: None)				
One grandchild			0.714**	(0.259)
Two grandchildren and above			0.687	(0.519)
The number of caregiving (Ref: None) # Gender				
One grandchild # male			−0.744*	(0.316)
Two grandchildren and above # male			−0.644	(0.610)
Age	−0.096	(0.137)	−0.100	(0.138)
Age-squared	0.001	(0.001)	0.001	(0.001)
Education level (Ref: Illiterate)				
Primary school	−0.093	(0.224)	−0.089	(0.224)
Middle school and above	0.038	(0.257)	0.041	(0.257)
Rural *hukou*	0.371+	(0.208)	0.369+	(0.208)
Marital status (Ref: Married)				
Divorced or separated	0.731	(0.734)	0.714	(0.733)
Widowed	−0.266	(0.234)	−0.268	(0.234)
Mother alive	0.047	(0.202)	0.053	(0.202)
Father alive	−0.172	(0.263)	−0.186	(0.263)
Smoking status	0.219	(0.176)	0.216	(0.176)
Drinks alcohol	−0.447**	(0.159)	−0.448**	(0.159)
Body mass index (Ref: Normal)				
Underweight	0.135	(0.341)	0.127	(0.340)
Overweight	0.521*	(0.202)	0.512*	(0.202)
Obesity	1.280***	(0.190)	1.273***	(0.190)
Number of ADL limitations	−0.110	(0.112)	−0.113	(0.112)
Number of IADL limitations	0.091	(0.071)	0.092	(0.070)
Self-reported health (Ref: Poor)				
Fair	−1.152*	(0.456)	−1.152*	(0.456)
Good	−1.205**	(0.453)	−1.206**	(0.454)
Very good	−1.153*	(0.495)	−1.167*	(0.495)
Excellent	−1.223*	(0.510)	−1.223*	(0.509)
Diagnosed diseases	0.791***	(0.085)	0.792***	(0.086)
Province	controlled		controlled	
_cons	3.111	(4.233)	3.251	(4.242)
*R*-squared	0.0954		0.0947	

*Notes*: ADL = activities of daily living; IADL = instrumental activity of daily living; *SE* = standard error. Standard errors, clustered at the household level, are shown in parentheses.

+*p* < .1. *p < .05. **p < .01. *** *p* < .001.

Compared with grandfathers, grandmothers may lose more from grandchild care. Looking after grandchildren with high intensity last year increases 0.89 years in biological aging for grandmothers, although increases only 0.11 (0.89–0.78) years in biological aging for grandfathers. The number of grandchildren care model shows that caring for one or two and more grandchildren is associated with greater biological aging for grandmothers than for grandfathers. Caring for one grandchild increases biological aging by 0.71 years for grandmothers, although it makes fewer change in biological aging for grandfathers (0.714–0.744 = −0.03)

### Spousal Care Buffers: Is the Relationship Between Grandchild Care and Biological Aging Dependent on Spouse Involvement?

Model 5 and Model 6 in Table 4 show the estimated association between caregiving types and biological aging, and its gender difference. Compared with those with no grandchild care, caring by self alone accelerates biological aging. For any grandparent, shared caregiving with spouse or caring by spouse alone is not significantly associated with greater biological aging.

**Table 4.** Estimating Biological Aging by Spousal Involvement in the Care

Variable	Model 5 (*n* = 3,384)		Model 6 (*n* = 3,384)	
	*β*	*SE*	*β*	*SE*
Caregiving types (Ref: No care)				
Caregiving alone	0.556*	(0.264)	0.681*	(0.312)
Caregiving by spouse alone	0.216	(0.305)	0.095	(0.376)
Caregiving by couples	0.274	(0.174)	0.726*	(0.304)
Male	0.359+	(0.207)	0.662*	(0.258)
Caregiving types (Ref: No care) # Gender				
Caregiving alone # male			−0.219	(0.546)
Caregiving by spouse alone # male			−0.010	(0.480)
Caregiving by couples # male			−0.794*	(0.350)
*R*-squared	0.0935		0.0950	

*Notes*: *SE* = standard error. Indicators for sociodemographic information, family context, health-related characteristics, and province as additional control variables are also included. Standard errors, clustered at the household level, are shown in parentheses.

+*p* < .1. *p < .05. **p < .01. ***p < .001.

A significant interaction between shared caregiving and gender is observed in Model 6. To be specific, regardless of gender, grandchild care by self alone is associated with greater biological aging, and grandchild care by spouse alone does not significantly alter respondent’s biological aging (Model 5). However, shared caregiving with spouse accelerates biological aging for grandmothers (by 0.726), and doesn’t accelerate biological aging for grandfathers (0.726–0.794 = −0.068), suggesting the gender difference between shared caregiving and biological aging.

### Robustness Test of the Results

In this section, we conduct four sensitivity tests to check the robustness of our results. First, being 5 years biologically younger at age 50 may be quite different from being 5 years biologically younger at age 70. Therefore, it may be meaningful to measure the proportional difference score of biological aging that indicates how much older or younger a person in biology than their chronological age, by subtracting chronological age from biological age and then dividing by chronological age. Results also show that middle-aged and older adults who provide excessive caregiving would accelerate biological aging (Supplementary Table 2).

Second, because grandparents may be providing care to both grandchildren and their own spouses, we thus reestimate all regression models by excluding sample in which spouses need to be cared for to mitigate the impact of dual burden of caregiving. Results are substantively similar to those presented above, suggesting that high intensity of grandchild care is accompanied by greater biological aging (Supplementary Table 3).

Third, we examine whether different living arrangement between grandparents and their adult children matters, especially when the younger generation leaves rural areas for employment in the cities, leaving children behind to be cared for by grandparents. This may further aggregate the psychological and biological burden on grandparents, without bringing about honor and achievement. Accordingly, we interact grandparenting type and living arrangement to explore the impact of living arrangements on biological aging (Supplementary Table 4). Results show that grandparenting without living with their adult children tends to have greater biological aging, and we hypothesize that this greater biological aging is due to difficulty in obtaining emotional and financial support from their adult children.

Finally, to minimize the effect of unobserved factors that do not vary change time, we use change regression model to examine the association of change in grandparenting and change in biological aging between 2011 and 2015 (Supplementary Table 5). Results show that compared to 2011 and 2015 noncaregivers, grandparents who cared for their grandchildren in 2011 or 2015 have a 0.90-year change in biological aging, and those who cared for their grandchildren both in 2011 and 2015 have the greatest change in biological aging, with an increase of 1.12 year, confirming the cumulative effect of grandchild care.

## Discussion

We draw motivation from the widely utilized role enhancement, role strain, and stress process model, key implication of which is that grandchild care is a conditionally emotional engagement, and excessive involvement would produce role tension, resulting in adverse biological consequences. Unlike existing studies that rely on self-reported health or a single biomarker, our study makes use of the rich biomarker set from CHARLS to analyze more accurately the potential biological consequence of grandchild care and its gender difference among Chinese middle-aged and older adults.

First, we find that high intensity of involvement, that is, a weekly 40 hr and above on grandchild care, is related to greater biological aging, whereas minimal or medium caregiving may not make difference, supporting our first research hypothesis. We suppose that caring for grandchildren more than 40 hr may bring more restriction than happiness or achievement, which is hypothesized to increase risk of biological aging. Grandparenthood is one of the most salient roles in later life due to the unique cultural value of family in China, and the high-intensity responsibilities of raising grandchildren bring them with negative experiences and considerable stress. Previous studies have shown that higher stress was significantly associated with cardiovascular disease (Steptoe & Kivimaki, 2012), and increased odds of having metabolic syndrome (Pyykkonen et al., 2010). In the case of grandchild care, when the role of grandparent is overburdened or exceeds one’s physical and mental capacities, caregiving tends to be stressful rather than rewarding and decides adverse biological outcomes, especially without living with their adult children.

Second, findings from gender-specific analyses confirm the impact of grandchild care are different for grandfathers and grandmothers, and grandmothers lose more from caregiving than grandfathers, supporting our second hypothesis. Societal expectations of the grandmothers’ role are often associated with nurturing emotional care and physical and labor-intensive grandchild care tasks, and females are usually seen as the primary caregiver. In accordance with gender norm, the obligation and burden of caring for grandchildren often falls disproportionately on grandmothers, which may invisibly add to the stress of the grandmother’s role, perhaps reflecting a pattern in the maldistributed family care between gender as in other developing countries (Horsfall & Dempsey, 2013). Furthermore, the gender difference between grandchild care and biological aging may be related to the form and nature of caregiving activities. Previous research has shown that grandmothers in China take on more intensive responsibilities (eg, feeding, bathing, and dressing) rather than the role of fun-seeker, playmate, and companion that are often assumed by grandfathers (Xie & Xia 2011). Such care responsibility causes grandmothers to bear more stress than grandfathers, being deprived of more leisure time (Horsfall & Dempsey, 2013), and leading to greater biological aging in grandmothers.

Third, our study also responds to previous appeals to take into account spousal support in research on health and aging (Monserud & Peek, 2014). The association between caregiving and biological aging is dependent on whether spouses participate in the care, especially for grandfathers. Shared caring with spouse could alleviate grandfather’s biological aging, but not reduce the negative effect of grandchild care on grandmothers, partly supporting our third hypothesis. This may be because, during the process of shared caring, grandfathers often act as playmates, rather than taking on more traditional daily caring responsibilities, which enhances their feelings of self-efficacy and self-esteem, improves their sense of self-worth, and strengthens close relationships with their extended family, all of which contribute positively to their psychological health and mitigate their biological aging. However, even if shared caring with spouse, grandmothers are still given more responsibility, and role stress and role burden do not actually decrease with their husbands’ involvement.

These findings together highlight the importance of gender context and spousal role in studying the biological effect of caregiving in China. In this study, the health disadvantage of caregiving is more pronounced in grandmothers whose caregiving conforms to the norm of role as family carer than grandfathers. This may be attributed to gender differences in the division of labor such that grandmothers provide more intensive care than grandfathers. Previous research has shown that females have consistently lower biological age than males, however, the gender difference decreases over time (Levine & Crimmins, 2018). Our results show that grandmothers experience higher tension and stress than grandfathers do in grandchild care, leading to greater biological aging. This finding may explain why biological age decreases less for females than males and why the gender gap in biological age is narrowing. In addition, spousal caring for grandchildren alters the association between grandchild care and biological aging for grandfathers, rather than grandmothers. This suggests that the stress, fatigue, and strain caused by taking care of grandchildren for grandmothers needs more family support to cope with.

Several limitations in the present study should be acknowledged. First, grandparents’ self-reports of caregiving activities could be subject to recall bias and social desirability bias. Meanwhile, although we measure caregiving by using the hours per week spent on grandchild care and the number of grandchildren care, it is still not explicitly defined due to the missing information on the details or types of caregiving activities, context-specific variables (eg, grandparent-grandchild relationships), and specific information about grandchildren being cared by the grandparent (eg, gender, age, and health status). The lack of information about caregiving responsibilities and contexts limits our ability to assess its relationship to biological outcomes. Future research is needed to design and implement better survey instruments to collect this information among Chinese older adults. Second, even though we use a change regression model, the limited follow-up information prevents a causal analysis of the impact of grandchild care on biological aging. Therefore, repeated measures of biological aging and grandchild care from multiple visits in the future will provide more important information in explaining the nature and direction of causality. Finally, quantification of biological aging itself is still active research, and there are other ways to quantify biological aging, such as PhenoAge, homeostatic dysregulation, perceived age, and epigenetic age. Using these methods to quantify aging and to examine its relationship to grandchild care is also warranted.

Despite these limitations, this study extends the literature on grandchild care to field of biology. Biological aging is a phenomenon that some individuals experience a faster aging rate compared with age-matched controls. Our study observes that high intensity of grandchild care is associated with greater biological aging among middle-aged and older adults from a nationally representative sample. Given the growing trend of grandparents caring for grandchildren and the important role of grandparent caregivers in China, providing grandchild care should be a way to cope with age-related role discontinuity or loss, rather than result in extra stress or burden for grandparents. Reducing the intensity of caregiving or increasing family or social support may attenuate the extent of biological aging. Our findings also suggest an urgent need to extend policies to alleviate the family responsibility for a large proportion of Chinese grandparents especially grandmothers who, in addition to facing their own aging challenges, have to care for grandchildren.

## Supplementary Material

igae059_suppl_Supplementary_Materials

## Data Availability

This study was not preregistered. The data sets generated and/or analyzed during the study are publicly available: https://charls.pku.edu.cn. All analytic codes used to generate results for this study are available by request from the first author for replication purposes.
