# Supplementary material for: The Association Between Grandchild Care and Biological Aging Among Middle-Aged and Older Adults in China
Source: Innov Aging. 2024 Jun 17;8(7):igae059. doi: 10.1093/geroni/igae059 (PMC11258899; doi:10.1093/geroni/igae059)
Supplement: igae059_suppl_Supplementary_Materials [file igae059_suppl_supplementary_materials.docx]

***Innovation in Aging* Supplementary Material: Xie & Wang. The association between grandchild care and biological aging among middle-aged and older adults in China.**

**Table S1. Selected aging biomarkers and their correlations with chronological age by gender**

| **Biomarker** | Males | | Females | |
| --- | --- | --- | --- | --- |
|  | Description | Corr | Description | Corr |
| Vascular function | Systolic Blood Pressure (mmHg) | 0.161^***^ | Systolic Blood Pressure (mmHg) | 0.303^***^ |
|  | Diastolic Blood Pressure (mmHg) | -0.152^***^ |  |  |
| Immune function | Platelets (10^9/L) | -0.107^***^ |  |  |
|  | Hematocrit (%) | -0.202^***^ |  |  |
|  |  |  | 1. Reactive Protein (mg/l) | 0.101^***^ |
|  |  |  | Mean Corpuscular Volume (fl) | 0.124^***^ |
| Metabolic function | Hemoglobin (g/dl) | -0.255^***^ |  |  |
|  | Triglycerides (mg/dl) | -0.188^***^ |  |  |
|  |  |  | LDL Cholesterol (mg/dl) | 0.122^***^ |
|  |  |  | Total Cholesterol (mg/dl) | 0.139^***^ |
|  |  |  | Glucose (mg/dl) | 0.117^***^ |
|  |  |  | Uric Acid (mg/dl) | 0.121^***^ |
|  | Creatinine (mg/dl) | 0.149^***^ | Creatinine (mg/dl) | 0.140^***^ |
|  | Blood Urea Nitrogen (mg/dl) | 0.130^***^ | Blood Urea Nitrogen (mg/dl) | 0.213^***^ |
|  | Cystatin C (mg/l) | 0.397^***^ | Cystatin C (mg/l) | 0.472^***^ |
|  | Glycated Hemoglobin (%) | 0.099^***^ | Glycated Hemoglobin (%) | 0.164^***^ |

*Notes*: ^+^ *p* < 0.1, ^*^ *p* < 0.05, ^**^ *p* < 0.01, ^***^ *p* < 0.001.

**Table S2. Alternative measures: Proportional difference score of biological aging**

| **Variable** | BAPS | | BAPS | |
| --- | --- | --- | --- | --- |
|  | *β* | SE | *β* | SE |
| Caregiving intensity (ref: No care) |  |  |  |  |
| Low | 0.002 | (0.004) |  |  |
| Medium | 0.004 | (0.004) |  |  |
| High | 0.008^**^ | (0.003) |  |  |
| The number of caregiving (ref: None) |  |  |  |  |
| One grandchild |  |  | 0.006^*^ | (0.003) |
| Two grandchildren and above |  |  | 0.006 | (0.005) |
| Age | -0.002 | (0.002) | -0.002 | (0.002) |
| Age-squared | 0.000 | (0.000) | 0.000 | (0.000) |
| Male | 0.006^+^ | (0.003) | 0.006^+^ | (0.003) |
| Education level (ref: Illiterate) |  |  |  |  |
| Primary school | -0.003 | (0.004) | -0.003 | (0.004) |
| Middle school and above | 0.000 | (0.004) | 0.000 | (0.004) |
| Rural *hukou* | 0.007^*^ | (0.003) | 0.007^*^ | (0.003) |
| Marital status (ref: Married) |  |  |  |  |
| Divorced or separated | 0.013 | (0.012) | 0.013 | (0.012) |
| Widowed | -0.004 | (0.004) | -0.005 | (0.004) |
| Mother alive | 0.001 | (0.004) | 0.001 | (0.004) |
| Father alive | -0.003 | (0.005) | -0.003 | (0.005) |
| Smoking status | 0.003 | (0.003) | 0.003 | (0.003) |
| Drinks alcohol | -0.007^**^ | (0.003) | -0.007^**^ | (0.003) |
| Body mass index (ref: Normal) |  |  |  |  |
| Underweight | 0.003 | (0.005) | 0.003 | (0.005) |
| Overweight | 0.009^**^ | (0.003) | 0.008^*^ | (0.003) |
| Obesity | 0.021^***^ | (0.003) | 0.021^***^ | (0.003) |
| Number of ADL limitations | -0.002 | (0.002) | -0.002 | (0.002) |
| Number of IADL limitations | 0.002 | (0.001) | 0.002 | (0.001) |
| Self-reported health (ref: Poor) |  |  |  |  |
| Fair | -0.018^*^ | (0.007) | -0.018^*^ | (0.007) |
| Good | -0.020^**^ | (0.007) | -0.020^**^ | (0.007) |
| Very good | -0.018^*^ | (0.008) | -0.018^*^ | (0.008) |
| Excellent | -0.020^*^ | (0.008) | -0.020^*^ | (0.008) |
| Diagnosed diseases | 0.013^***^ | (0.001) | 0.013^***^ | (0.001) |
| Province | controlled | | controlled | |
| _cons | 0.056 | (0.072) | 0.059 | (0.072) |
| *n* | 3,384 | | 3,384 | |
| R-squared | 0.0936 | | 0.0930 | |

*Notes*: BAPS = proportional difference score of biological aging = (biological age - chronological age)/chronological age; ADL = activities of daily living; IADL = instrumental activity of daily living. Standard errors, clustered at the household level, are shown in parentheses.

^+^ *p* < 0.1, ^*^ *p* < 0.05, ^**^ *p* < 0.01, ^***^ *p* < 0.001.

**Table S3. Excluding cases whose spouses need to be cared for**

| **Variable** | Biological aging | | Biological aging | |
| --- | --- | --- | --- | --- |
|  | *β* | SE | *β* | SE |
| Caregiving intensity (ref: No care) |  |  |  |  |
| Low | 0.113 | (0.274) |  |  |
| Medium | 0.321 | (0.265) |  |  |
| High | 0.516^*^ | (0.210) |  |  |
| The number of caregiving (ref: None) |  |  |  |  |
| One grandchild |  |  | 0.366^*^ | (0.181) |
| Two grandchildren and above |  |  | 0.363 | (0.324) |
| Age | -0.062 | (0.153) | -0.067 | (0.153) |
| Age-squared | 0.000 | (0.001) | 0.000 | (0.001) |
| Male | 0.224 | (0.231) | 0.219 | (0.231) |
| Education level (ref: Illiterate) |  |  |  |  |
| Primary school | -0.067 | (0.246) | -0.064 | (0.246) |
| Middle school and above | -0.127 | (0.283) | -0.122 | (0.283) |
| Rural *hukou* | 0.274 | (0.233) | 0.271 | (0.233) |
| Marital status (ref: Married) |  |  |  |  |
| Divorced or separated | 0.702 | (0.746) | 0.691 | (0.745) |
| Widowed | -0.429^+^ | (0.244) | -0.431^+^ | (0.243) |
| Mother alive | 0.075 | (0.224) | 0.082 | (0.224) |
| Father alive | -0.002 | (0.292) | -0.013 | (0.292) |
| Smoking status | 0.331 | (0.201) | 0.335^+^ | (0.201) |
| Drinks alcohol | -0.586^***^ | (0.177) | -0.589^***^ | (0.177) |
| Body mass index (ref: Normal) |  |  |  |  |
| Underweight | 0.115 | (0.374) | 0.107 | (0.375) |
| Overweight | 0.557^*^ | (0.224) | 0.557^*^ | (0.224) |
| Obesity | 1.328^***^ | (0.213) | 1.328^***^ | (0.213) |
| Number of ADL limitations | -0.072 | (0.139) | -0.076 | (0.139) |
| Number of IADL limitations | 0.028 | (0.077) | 0.029 | (0.077) |
| Self-reported health (ref: Poor) |  |  |  |  |
| Fair | -1.085^*^ | (0.495) | -1.078^*^ | (0.496) |
| Good | -1.138^*^ | (0.485) | -1.135^*^ | (0.486) |
| Very good | -1.205^*^ | (0.531) | -1.222^*^ | (0.533) |
| Excellent | -0.837 | (0.546) | -0.835 | (0.547) |
| Diagnosed diseases | 0.807^***^ | (0.095) | 0.806^***^ | (0.095) |
| Province | controlled | | controlled | |
| _cons | 2.205 | (4.697) | 2.388 | (4.705) |
| *n* | 2,738 | | 2,738 | |
| R-squared | 0.1007 | | 0.1001 | |

*Notes*: Of the 3,384 respondents we analyzed, 2,714 were married and 670 were spouseless. Of those with spouses, 547 had spouses who reported loss of self-care ability and 99 were unable to identify their spouse’s health status. Therefore, the sample size for this part was reduced to 2738 (2714-547-99+670), and these respondents did not provide care to their own spouses. ADL=activities of daily living; IADL = instrumental activity of daily living. Standard errors, clustered at the household level, are shown in parentheses.

^+^ *p* < 0.1, ^*^ *p* < 0.05, ^**^ *p* < 0.01, ^***^ *p* < 0.001.

**Table S4. The association of grandchild care and biological aging by living arrangement**

| **Variable** | Biological aging | | Biological aging | |
| --- | --- | --- | --- | --- |
|  | *β* | SE | *β* | SE |
| Caregiving intensity (ref: No care) |  |  |  |  |
| Low | 0.068 | (0.364) |  |  |
| Medium | 0.320 | (0.360) |  |  |
| High | 0.909^***^ | (0.267) |  |  |
| The number of caregiving (ref: None) |  |  |  |  |
| One grandchild |  |  | 0.433^+^ | (0.227) |
| Two grandchildren and above |  |  | 1.055^*^ | (0.464) |
| Living with adult children | 0.089 | (0.216) | 0.086 | (0.216) |
| Caregiving intensity (ref: No care) # Living with adult children |  |  |  |  |
| Low # yes | 0.174 | (0.506) |  |  |
| Medium # yes | -0.210 | (0.469) |  |  |
| High # yes | -0.730^*^ | (0.371) |  |  |
| The number of caregiving (ref: None) # Living with adult children |  |  |  |  |
| One grandchild # yes |  |  | -0.186 | (0.316) |
| Two grandchildren and above # yes |  |  | -1.338^*^ | (0.603) |
| Age | -0.106 | (0.137) | -0.110 | (0.138) |
| Age-squared | 0.001 | (0.001) | 0.001 | (0.001) |
| Male | 0.364^+^ | (0.204) | 0.348^+^ | (0.204) |
| Education level (ref: Illiterate) |  |  |  |  |
| Primary school | -0.065 | (0.223) | -0.064 | (0.223) |
| Middle school and above | 0.040 | (0.257) | 0.049 | (0.257) |
| Rural *hukou* | 0.397^+^ | (0.208) | 0.393^+^ | (0.208) |
| Marital status (ref: Married) |  |  |  |  |
| Divorced or separated | 0.794 | (0.733) | 0.780 | (0.737) |
| Widowed | -0.255 | (0.233) | -0.272 | (0.233) |
| Mother alive | 0.053 | (0.202) | 0.049 | (0.202) |
| Father alive | -0.172 | (0.263) | -0.158 | (0.264) |
| Smoking status | 0.192 | (0.176) | 0.202 | (0.176) |
| Drinks alcohol | -0.461^**^ | (0.160) | -0.464^**^ | (0.160) |
| Body mass index (ref: Normal) |  |  |  |  |
| Underweight | 0.138 | (0.341) | 0.137 | (0.340) |
| Overweight | 0.521^**^ | (0.202) | 0.502^*^ | (0.202) |
| Obesity | 1.281^***^ | (0.190) | 1.256^***^ | (0.190) |
| Number of ADL limitations | -0.113 | (0.112) | -0.112 | (0.112) |
| Number of IADL limitations | 0.092 | (0.070) | 0.090 | (0.070) |
| Self-reported health (ref: Poor) |  |  |  |  |
| Fair | -1.169^*^ | (0.457) | -1.150^*^ | (0.457) |
| Good | -1.215^**^ | (0.455) | -1.198^**^ | (0.455) |
| Very good | -1.154^*^ | (0.496) | -1.154^*^ | (0.495) |
| Excellent | -1.222^*^ | (0.511) | -1.218^*^ | (0.510) |
| Diagnosed diseases | 0.785^***^ | (0.086) | 0.789^***^ | (0.086) |
| Province | controlled | | controlled | |
| _cons | 3.568 | (4.230) | 3.723 | (4.242) |
| *n* | 3,384 | | 3,384 | |
| R-squared | 0.0950 | | 0.0945 | |

*Notes*: ADL = activities of daily living; IADL = instrumental activity of daily living. Standard errors, clustered at the household level, are shown in parentheses.

^+^ *p* < 0.1, ^*^ *p* < 0.05, ^**^ *p* < 0.01, ^***^ *p* < 0.001.

**Table S5. Grandparenting and change in biological aging**

| **Variable** | Change in biological aging | |
| --- | --- | --- |
|  | *β* | SE |
| Grandparenting (ref: Never care) |  |  |
| Grandparenting in 2011 or 2015 | 0.896^*^ | (0.441) |
| Grandparenting from 2011 to 2015 | 1.118^*^ | (0.476) |
| Age | -0.463 | (0.387) |
| Age-squared | 0.004 | (0.003) |
| Male | 0.412 | (0.556) |
| Education level (ref: Illiterate) |  |  |
| Primary school | -0.812 | (0.497) |
| Middle school and above | -0.588 | (0.641) |
| Rural *hukou* | -0.564 | (0.636) |
| Marital status (ref: Married) |  |  |
| Divorced or separated | 7.602^*^ | (3.045) |
| Widowed | -0.932^+^ | (0.523) |
| Mother alive | 0.522 | (0.529) |
| Father alive | -0.803 | (0.732) |
| Smoking status | -0.297 | (0.462) |
| Drinks alcohol | 0.785^+^ | (0.445) |
| Body mass index (ref: Normal) |  |  |
| Underweight | -0.352 | (0.782) |
| Overweight | -0.241 | (0.481) |
| Obesity | 0.522 | (0.479) |
| Number of ADL limitations | -0.221 | (0.317) |
| Number of IADL limitations | 0.368^*^ | (0.161) |
| Self-reported health (ref: Poor) |  |  |
| Fair | -1.178 | (1.001) |
| Good | -1.414 | (0.962) |
| Very good | -0.348 | (1.073) |
| Excellent | -0.593 | (1.095) |
| Diagnosed diseases | 0.067 | (0.196) |
| Province | controlled | |
| _cons | 12.882 | (12.326) |
| *n* | 738 | |
| R-squared | 0.1374 | |

*Notes*: Change in biological aging =biological aging_2015_ **-** biological aging_2011_; ADL = activities of daily living; IADL = instrumental activity of daily living. Standard errors, clustered at the household level, are shown in parentheses.

^+^ *p* < 0.1, ^*^ *p* < 0.05, ^**^ *p* < 0.01, ^***^ *p* < 0.001.
